# Wonders of Surgery

**Published:** 1890-12

**Authors:** 


### Editorial, Etc.

WONDERS OF SURGERY.—The wonders of surgical success continue to multiply. The doctors are now sure that the famous operation of skin grafting has succeeded. The flesh of the dog and the boy have grown together. About ten days ago an inch of the tibia bone of John Gethius, aged fourteen, was removed, and a corresponding length of the bone of a dog's foreleg was put in its place. The ligaments of the dog's and boys' legs were sewn together, and since that time the boy and the dog, which has been named Charity, have been lying side by side, waiting for the bones to unite. The hiatus in the shin bone of the boy will now be filled by the transplanted shin of the dog.

At the Roosevelt Hospital two extraordinary operations have been performed for cancer of the throat. Ex-Senator Peter Ward, of Newburg, had his entire tongue cut out by Dr. Charles McBirney. The doctor cannot say that the operation will be a success until some time, or that the danger of so critical a strain is over. At the same institution Dr. W. T. Bull recently removed a patient's larynx for cancer of that organ. He has since steadily improved. The surgeon is now to provide him with an artificial speaking apparatus. A silver tube slightly curved has been made to enter the opening left where the larynx had been. This passes down to the cut top of the windpipe. The tube was made in such a way that another tube, smaller and more curved, connecting with it, put in separately, could be passed into the same opening and upward into the back part of the mouth, partly filling the cavity occupied formerly by the larynx, and connecting the first tube with the interior of the mouth. After these tubes were put in place the tracheotomy tube was taken out of the windpipe and the orifice closed; then the patient could breathe through the mouth.

The surgeon then procured a hollow plug to fit the tube that runs into the mouth. In this a reed like the tongue of a harmonica vibrates in a distinct tone and note when the outer orifice is closed, and the air drawn in at the mouth, through the two joined tubes, into the lungs, and from the lungs expelled in the same manner. But it is not an easy matter for art to replace nature, and, as might be expected, the doctor at first experienced some trouble in fitting this reed. But the patient can now talk almost as well as ever he could, though his voice is a monotone, taking constantly the pitch of the reed. Should his voice fail him new reeds can be put in at any time. After the success of the new shin and the new larynx efforts there seems nothing in the range of surgical possibilities that cannot now be accomplished.

Since the possibilities in skin-grafting first became evident to surgeons a great extension in plastic surgery has taken place. The bones of men's fingers have been stuck to their noses when the nasal bones have been removed for some sufficient cause. Union between the finger and the nose has followed; then they have been cut apart by the surgeon's knife, leaving a piece of finger-bone to act vicariously for the lost nasal one.

This bone transplanting from one part of the human body to another has been so successful as to suggest the substitution of the bone of one of the lower vertebrate animals under practically similar conditions. So far as is known, the first operation of this kind was performed a short time ago in the Charities Hospital, on Blackwell's Island, by Dr. Phelps, assisted by Dr. Kelly.

The case was that of a boy of fourteen. The larger bone of the two which connect the knee and ankle joints was wanting for an inch of its length near its lower end. Efforts had been made in a previous operation to spread the bone between the points of separation, so that a healthy and strong enough backing should grow naturally on it, but without success. The boy was healthy, and, it seemed to the eminent surgeons, a suitable case for trying the experiment of substituting an inch of dog's bone for the hiatus in the human one.

The right forepaw of a dog was painlessly removed under anæsthetics and an inch of the lower end of the skin-bone was cut off without detaching the muscles and with great care in order to preserve the nutrient artery of the bone intact.

Then Dr. Phelps cut down on the boy's shin-bone so as to expose the gap in it. Then the inch of dog's bone was let into this space and fastened at both ends by silver wire. Both boy and dog had previously had their contiguous limbs rendered immovable by plaster of Paris bandages.

Dog and boy must remain together until there has been complete union established between the circulations of the adjoining bones. In order to accomplish this object the muscles and blood vessels of the dog's bone have been preserved as far as possible. This is to keep the bone alive until it has been invaded by millions of human capillaries which ought to spread over it from the surrounding tissues in the boy's leg.

At the end of some time it is probable that this union will so far have taken place as to allow the muscles and blood vessels connecting the bone with the dog to be removed. Then the patients will be cut apart and the healing process of nature allowed to go on independently in each case.

The boy was in excellent health two days after the operation, and there is no doubt about his recovery. Unfortunately, the dog's condition is not so favorable. In order to keep him sufficiently immobile to permit of the success of the operation, his body was put in a plaster of Paris jacket. This enforced restraint has told on his health.

---

